# A Comparison of *EGFR* Mutation Testing Methods in Lung Carcinoma: Direct Sequencing, Real-time PCR and Immunohistochemistry

**DOI:** 10.1371/journal.pone.0043842

**Published:** 2012-08-27

**Authors:** Bárbara Angulo, Esther Conde, Ana Suárez-Gauthier, Carlos Plaza, Rebeca Martínez, Pilar Redondo, Elisa Izquierdo, Belén Rubio-Viqueira, Luis Paz-Ares, Manuel Hidalgo, Fernando López-Ríos

**Affiliations:** 1 Laboratorio de Dianas Terapéuticas, Faculty of Medicine, Centro Integral Oncológico “Clara Campal”, Hospital HM Universitario Sanchinarro, Universidad San Pablo-CEU, Madrid, Spain; 2 Gastrointestinal Cancer Clinical Research Unit, Spanish National Cancer Research Centre, Madrid, Spain; 3 Department of Oncology, Instituto de Biomedicina de Sevilla (IBIS) & Hospital Universitario Virgen del Rocío, Sevilla, Spain; The Chinese University of Hong Kong, Hong Kong

## Abstract

The objective of this study is to compare two *EGFR* testing methodologies (a commercial real-time PCR kit and a specific *EGFR* mutant immunohistochemistry), with direct sequencing and to investigate the limit of detection (LOD) of both PCR-based methods. We identified *EGFR* mutations in 21 (16%) of the 136 tumours analyzed by direct sequencing. Interestingly, the Therascreen EGFR Mutation Test kit was able to characterize as wild-type one tumour that could not be analyzed by direct sequencing of the PCR product. We then compared the LOD of the kit and that of direct sequencing using the available mutant tumours. The kit was able to detect the presence of a mutation in a 1% dilution of the total DNA in nine of the 18 tumours (50%), which tested positive with the real-time quantitative PCR method. In all cases, *EGFR* mutation was identified at a dilution of 5%. Where the mutant DNA represented 30% of the total DNA, sequencing was able to detect mutations in 12 out of 19 cases (63%). Additional experiments with genetically defined standards (EGFR ΔE746-A750/+ and EGFR L858R/+) yielded similar results. Immunohistochemistry (IHC) staining with exon 19-specific antibody was seen in eight out of nine cases with E746-A750del detected by direct sequencing. Neither of the two tumours with complex deletions were positive. Of the five L858R-mutated tumours detected by the PCR methods, only two were positive for the exon 21-specific antibody. The specificity was 100% for both antibodies. The LOD of the real-time PCR method was lower than that of direct sequencing. The mutation specific IHC produced excellent specificity.

## Introduction

In 2004, it was discovered that the reason why some patients with adenocarcinomas of the lung responded in spectacular form to treatment with tyrosine kinase inhibitors (TKIs) of *EGFR* was specifically due to the existence of activating mutations of this gene [1]–[3]. This discovery caused a wave of enthusiasm in the therapy of such an aggressive tumour. Study of the mutational state of *EGFR* became a matter of urgent necessity in patients with adenocarcinomas of the lung.

The most commonly used methodology for this purpose has been, and will probably continue to be, direct sequencing of PCR products. The main drawbacks of this method are its low sensitivity (20–50%) and the significant risk of contamination involved in handling post-PCR products. Nevertheless, useful and ingenious alternatives have been developed but, despite their proven sensitivity, they have never become popular [4]–[7]. Furthermore, recent advances in molecular techniques have enabled the development of more sensitive methods for detecting mutations with real-time quantitative PCR, using specific probes or amplified refractory mutation system (ARMS™) technology [8]–[10]. Most recently, the development of *EGFR* mutant-specific antibodies for immunohistochemistry (IHC) has presented a new method for consideration [11]–[21]. Seven years after this major discovery, there is still no standardized test approved by the US Food and Drug Administration and the current diversity of methods for conducting this test is creating serious logistical problems worldwide.

In this article, we present our experience in the study of *EGFR* mutations, comparing direct sequencing, the gold standard, with a commercial real-time quantitative PCR kit (Therascreen EGFR Mutation Test) and IHC; as well as determining the limit of detection (LOD) of both PCR-based methods.

## Methods

Written informed consent was obtained from all participants involved. We obtained ethics approval from the ethics committee at the institution where samples were analyzed (Grupo Hospital de Madrid).

One hundred and thirty six formalin-fixed paraffin-embedded (FFPE) tumours from patients diagnosed with non-small cell lung carcinoma (NSCLC) were collected from our files. All patients had been tested as part of standard clinical practice. Patient and tumour characteristics, such as age, gender, smoking status, histology and tumour sample type, are summarized in Table 1. The material available for all tumours was tissue blocks. Of all the samples analyzed, 43 were bronchoscopic biopsies (31.6%), 7 core-needle biopsies (CNBs) (5.2%), and 86 surgical specimens (63.2%). Before DNA extraction, representative sections were stained with haematoxylin and eosin (H&E) and tumours were reviewed by two pathologists (EC and FL-R) and histologically classified according to the 2004 WHO criteria. Histological characteristics of the tumours included in the mutational analysis of the *EGFR* gene were as follows: 32 (23.5%) carcinomas NOS, 14 (10.3%) squamous cell carcinomas (SCC), 87 (64%) adenocarcinomas (AC), and three (2.2%) large cell carcinoma (LCC). Moreover, the percentages of tumour cells and extracellular mucin, if there was a relevant amount (more than 50% of the tumour), or lymphocyte inflammation (more than 10% of lymphocytes at 20× magnification) were assessed. This was because it is well known that the sensitivity of PCR-based assays is influenced by the presence of non-tumour material, such as mucin, non-neoplastic normal cells or lymphocytes [22].

**Table 1:** Clinicopathologic features of the tumours included in the *EGFR* mutation analysis.

		Whole series	*EGFR* mutant	*EGFR* wild-type	
		n (%)	n (%)	n (%)	p
		136# (x)	21 (16)	112 (84)	<0.001**
Age (yr)*	Mean ±SD	60.1±8.9	62.5±9.09	59.3±11.5	0.405
	Median	61	62.5	59	
	Range	31–84	43–84	31–81	
Sex	Male	83 (61)	5 (23.8)	76 (67.9)	<0.001**
	Female	53 (39)	16 (76.2)	36 (32.1)	
Tobacco smoking status*	Ex-smoker	42 (47.2)	3 (18.8)	38 (53.5)	<0.001**
	Current smoker	29 (32.6)	1 (6.2)	27 (38)	
	Never smoker	18 (20.2)	12 (75)	6 (8.5)	
Histology	Carcinoma NOS	32 (23.5)	4 (19)	27 (24.1)	0.781
	Adenocarcinoma	87 (64)	17 (81)	68 (60.7)	0.088
	•Lepidic component	22 (18.5)	6 (31.6)	16 (16.3)	0.125
	Squamous cell carcinoma	14 (10.3)	0	14 (12.5)	0.125
	Large cell carcinoma	3 (2.2)	0	3 (2.7)	1
Tumour sample	Bronchoscopic biopsies	43 (31.6)	8 (38.1)	34 (30.3)	0.484
	Core-needle biopsies	7 (5.2)	0	5 (4.5)	0.324
	Surgical specimens	86 (63.2)	13 (61.9)	73 (65.2)	0.773

# The *EGFR* mutation analysis by direct sequencing was not evaluable for three of the tumours included in the series.

* Unknown characteristic for some of the tumours included in the *EGFR* mutation analysis.

** Statistically significant p<.05.

The pre-analytical phase of the PCR procedures has been described previously [22]. Briefly, macrodissection of the tumour from the paraffin block was carried out to enrich the final proportion of tumour DNA. Macrodissection was performed to guarantee at least 30% tumour in all cases in which there was sufficient material available for analysis. The DNA extraction was performed with QIAamp™ DNA FFPE Tissue kit and automated on the QIAcube robot (QIAGEN, Valencia, CA, USA), as previously described and according to the manufacturer’s instructions [22].

The presence of *EGFR* mutations was determined by three methods. Firstly, as part of standard clinical practice, we performed the mutation analysis by direct sequencing (the current gold standard). Retrospectively, we used a real-time quantitative PCR based-approach, and IHC. Mutation screening of exons 18, 19, 20 and 21 of the *EGFR* gene was carried out by PCR amplification followed by automatic direct sequencing as previously described [23]. Exons 18–21 of the *EGFR* gene were amplified in duplicate and all variants were confirmed by re-sequencing independent PCR products. All sequencing reactions were performed in both forward and reverse directions and all the electropherograms were analyzed by visual inspection by a highly experienced observer (>1000 sequences/year, BA).

Secondly, the presence of *EGFR* mutations was determined with the Therascreen EGFR Mutation Test kit (Qiagen Manchester Ltd., Manchester, UK) which is designed to detect the most commonly reported *EGFR* mutations: 19 deletions in exon 19, three insertions in exon 20, and the point mutations G719X (exon 18), S768I and T790 M (exon 20), and L858R and L861Q (exon 21). The kit combines two technologies (ARMS™, Astrazeneca, and Scorpions™, Qiagen Manchester Ltd.) to detect these mutations by real-time quantitative PCR [24]–[26]. Allele specific amplification was achieved with the ARMS™ technology and Scorpions™ was used as a fluorescent signalling system to detect the PCR products. The analysis was performed according to the manufacturer’s instructions using an ABI PRISM 7300 (Applied Biosystems Inc, Foster City, CA, USA).

Thirdly, we performed IHC with two mutation-specific anti-EGFR antibodies: E746-A750del (exon 19) (6B6, 1∶25 dilution; Cell Signaling Technology Inc., Danvers, MA, USA) and L858R (exon 21) (43B2, 1∶100 dilution; Cell Signaling Technology Inc.). Fully automated IHC was conducted in a Benchmark XT (Ventana Medical Systems Inc., Tucson, AZ), according to the manufacturer’s instructions. Sections were counterstained with haematoxylin. Immunostaining was evaluated by two different pathologists (EC and FL-R), using criteria based on published cut-offs. The intensity of the cytoplasmic and/or membrane staining, as well as the percentage of positive cells, was recorded. Staining intensity was scored from 0 to 3+, as follows: 0 if tumour cells had complete absence of staining or faint staining intensity in <10%; 1+ if >10% of tumour cells had faint staining; 2+ if tumour cells had moderate staining; and 3+ if tumour cells had strong staining [12], [13]. Accordingly, we classified scores of 0 and 1+ as negative and scores of 2+ and 3+ as positive. To assess the sensitivity and specificity of IHC, we compared these results with those of direct sequencing and real-time quantitative PCR.

Next, we assessed the LOD of both PCR-based methods by two approaches. Firstly, using two commercial *EGFR* genetically defined standards [EGFR ΔE746-A750/+ gDNA Package and EGFR L858R/+ gDNA Package, (Horizon Diagnostics, Cambridge, UK)], we prepared a series of dilutions to give different final mutant allele frequencies: 1%, 3% and 5% for the Therascreen EGFR Mutation Test kit, and 10%, 20%, 25% and 30% for direct sequencing. Secondly, using all *EGFR* positive tumours with DNA available for further studies, we selected pairs of mutant and wild-type tumours that had a similar percentage of tumour cells and were equivalent in terms of the quality and quantity of the DNA extracted. Based on our previous experience [22], serial dilutions of DNA were performed by mixing DNA extracted from a mutant tumour into DNA extracted from a wild-type tumour, to give a final proportion of mutant DNA relative to wild-type DNA of 1%, 3% and 5% for the Therascreen EGFR Mutation Test Kit; and 10%, 20%, 25% and 30% for direct sequencing.

Finally, we wanted to determine whether the mutational status of the *EGFR* gene was associated with any of the clinicopathologic features of the tumours evaluated. Accordingly, frequencies were compared either by Fisher’s exact test or by the *X* ^2^ contingency test (U Mann-Whitney non-parametric test, in the case of the age). Differences of p<0.05 were considered statistically significant. Analyses were performed using the SPSS/PASW program, version 18.0 (SPSS Inc, Chicago, IL).

## Results

Regarding the pre-analytical phase in the study of *EGFR* mutations, it is important to note that macrodissection was performed on 22 of the 136 tumours analyzed (16%); 95.5% of the macrodissected samples were surgical specimens. The availability of material for molecular analysis was limited in the case of bronchoscopic biopsies and CNBs. For this reason, macrodissection was possible in only one bronchoscopic biopsy. After DNA extraction, at least 100 ng/µl of DNA were obtained from 65% of the samples. Of the samples that yielded a lower DNA concentration, 92% were small biopsies (bronchoscopic biopsies or CNBs).

*EGFR* exons 18–21 were successfully analyzed in 133 tumours by PCR amplification followed by direct sequencing. The three samples (one bronchoscopic biopsy and two CNBs) that could not be evaluated by direct sequencing were characterized by the poor quality of the extracted DNA (ratio A_260_/A_230_∼1.6, ∼0.2 and ∼0.7, respectively). Direct sequencing identified *EGFR* mutations in 21 (16%) of the analyzed tumours. All of them were previously described changes, either amino acid substitutions in exon 21, in-frame deletions in exon 19, and in-frame insertions in exon 20. The frequencies of the different types of *EGFR* mutations were as follows: 13 out of 21 (62%) exon 19 deletions, including the typical deletion of 15 nucleotides E746-A750 and atypical and/or complex deletions, five out of 21 (24%) exon 21 L858R point mutations, and three out of 21 (14%) exon 20 insertions. As indicated in Table 1, *EGFR* mutations were more common in females and in never smokers (p<0.001). Although the relationship was not statistically significant, *EGFR* mutations occurred also more frequently in ACs compared to other histological types. Table 2 summarizes the *EGFR* mutations detected by direct sequencing and interesting findings related to the pre-analytical phase of gene mutation analysis. Most of the mutated tumours had at least 40% of tumour cells, and enough DNA quantity and quality ([DNA]>100 ng/ul, A_260_/A_280_∼2). Interestingly, it is important to note that the *EGFR* detection rates for the surgical specimens and for small biopsies (bronchoscopic and CNBs) were similar: 13 out of 86 (15%) and eight out of 50 (16%), respectively.

**Table 2:** Mutations in the *EGFR* Gene Detected by Direct Sequencing and Tumour.

*EGFR* Mutation		Tumour characteristics				
Exon	Change	Sample type	% Tumour*	Macrodissection	[DNA](ng/µl)	A_260_/A_230_
19	E746-A750 del	Surgical specimen	80	No	870	2.37
19	E746-A750 del	Bronchoscopic biopsy	10	No	28	1.7
19	E746-A750 del	Bronchoscopic biopsy	25	No	12	1.57
19	E746-A750 del	Surgical specimen	50	No	810	2.13
19	E746-A750 del	Surgical specimen	60 (25)	Yes	150	1.8
19	E746-A750 del	Surgical specimen	85	No	2530	2.09
19	E746-A750 del	Bronchoscopic biopsy	40	No	98	2.27
19	E746-A750 del	Bronchoscopic biopsy	95	No	25	1.5
19	E746-A750 del	Bronchoscopic biopsy	40	No	17	2.5
19	L747-A750>P del	Surgical specimen	40	No	590	2.1
19	L747-P753>S del	Surgical specimen	65	No	947	2.3
19	E746-S752>V del	Bronchoscopic biopsy	50	No	20	2.03
19	L747-A750>P del	Bronchoscopic biopsy	65	No	14	2.3
21	L858R	Surgical specimen	80	No	33.5	1.98
21	L858R	Surgical specimen	70	No	685	2.16
21	L858R	Surgical specimen	85 (20)	Yes	245	2.23
21	L858R	Surgical specimen	25 (5)	Yes	336	2.25
21	L858R	Bronchoscopic biopsy	30	No	14.5	1.5
20	D770-N771 insSVD	Surgical specimen	90	No	630	2.4
20	V769-D770 insASV	Surgical specimen	80	No	362	2.2
20	V769-D770 insASV	Surgical specimen	40	No	995	2.23

Characteristics Related to the Pre-analytical Phase.

del, deletion.

ins, insertion.

* In parenthesis, the initial percentage of tumor cells for macrodissected samples.

**Figure 1:**
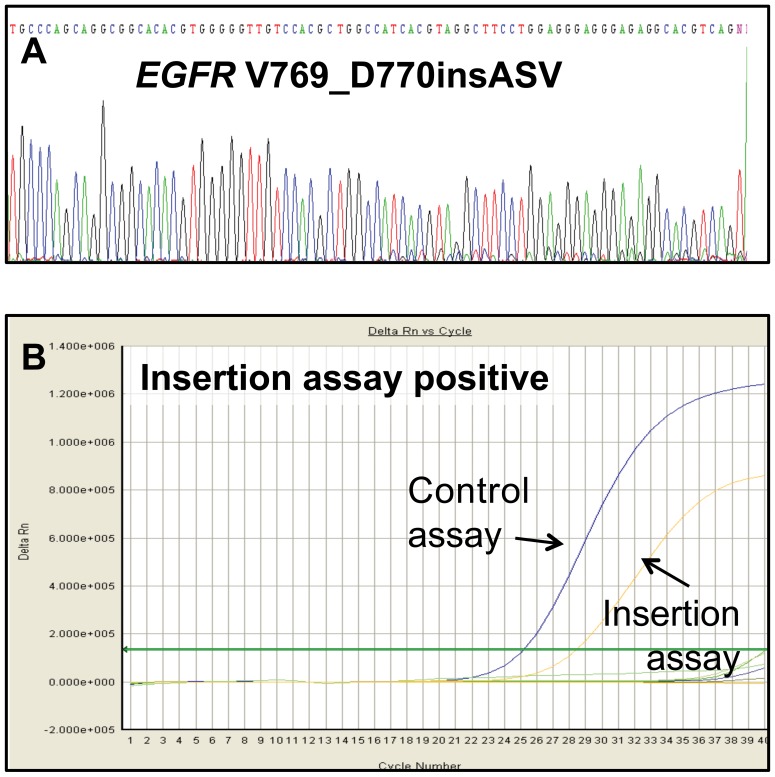
*EGFR* mutational results for (A) direct sequencing, and (B) the Therascreen *EGFR* Mutation Test Kit. The electropherogram depicts an *EGFR* insertion V769_D770 insASV in exon 20 (sequence obtained from reverse primer) and the amplification graph corresponds to the same tumour analyzed with the Therascreen EGFR Mutation Test Kit. The low signal intensity corresponding to the mutated sequence for the *EGFR* deletion and insertion can be confused with background noise, which makes interpretation more difficult, while the interpretation of the result of the Therascreen EGFR Mutation Test was unequivocal.

Sufficient DNA was not available for additional studies for six of the tumours included in the *EGFR* mutation screening by direct sequencing. These were two mutants, both with E746-A750 deletion, and four wild-type tumours. *EGFR* mutational status obtained with direct sequencing was confirmed with the Therascreen EGFR Mutation Test Kit in 126 of the samples included in the comparative study: 18 mutated and 108 wild-type tumours. Table 3 summarizes the sensitivity, specificity, positive predictive value (PPV), negative predictive value (NPV) and accuracy of the real-time quantitative PCR-based method. Interestingly, compared with direct sequencing, the sensitivity of the Therascreen EGFR Mutation Test kit was 95% because one ‘false’ negative result was obtained with the real-time quantitative PCR method: one tumour with an insertion in exon 20 (D770_n771insSVD), detected by direct sequencing, was tested as negative by the kit because the insertions assay is not designed to detect this type of alteration. If we consider only the mutations that the kit can detect, the sensitivity was 100%. On the other hand, the Therascreen EGFR Mutation Test kit allowed the characterization as wild-type of one of the three tumours that could not be analyzed by direct sequencing. Interestingly, this sample exhibited the best DNA quality of the three samples (A_260_/A_230_ ∼1.6). Regarding the post-analytical phase (interpretation), it is important to note that for one *EGFR* mutated sample (exon 20 insertion), the direct sequencing result was not clear and experience in sequence interpretation was required. The low signal intensity corresponding to the mutated sequence could be confused with background and could lead to a false negative result. However, the interpretation of the real-time quantitative PCR was unequivocal (Figure 1). This sample was a surgical specimen with a high percentage of tumour cells (80%) and, therefore, macrodissection was not performed.

**Table 3:** Detection Capabilities for the Therascreen EGFR Mutation Test kit *versus* Direct Sequencing.

	Direct sequencing					
*EGFR* mutations	Sensitivity (%)	95% CI	Specificity (%)	PPV (%)	NPV (%)	Accuracy (%)
Exon 19 deletions	100	100–100	100	100	100	100
L858R	100	100–100	100	100	100	100
Exon 20 insertions	67.7	58.5–74.9	100	100	99.2	99.2
All mutations	94.7	90.9–98.6	100	100	99.1	99.2

CI, confidence interval.

PPV, positive predictive value.

NPV, negative predictive value.

We then compared the LOD of the kit and that of direct sequencing using two *EGFR* genetically defined standards (ΔE746-A750/+ gDNA Package and L858R/+ gDNA Package), and the 19 *EGFR* mutant tumours for which an adequate quantity and quality of DNA was available. The Therascreen EGFR Mutation Test Kit was able to positively detect the E746-A750 deletion in a sample containing 5% down to 1% mutant allele frequency, and the L858R point mutation in a sample containing 5% down to 3% mutant allele frequency. Direct sequencing correctly identified the presence of both mutations in a sample containing 30% down to 20% mutant allele frequency.

**Figure 2:**
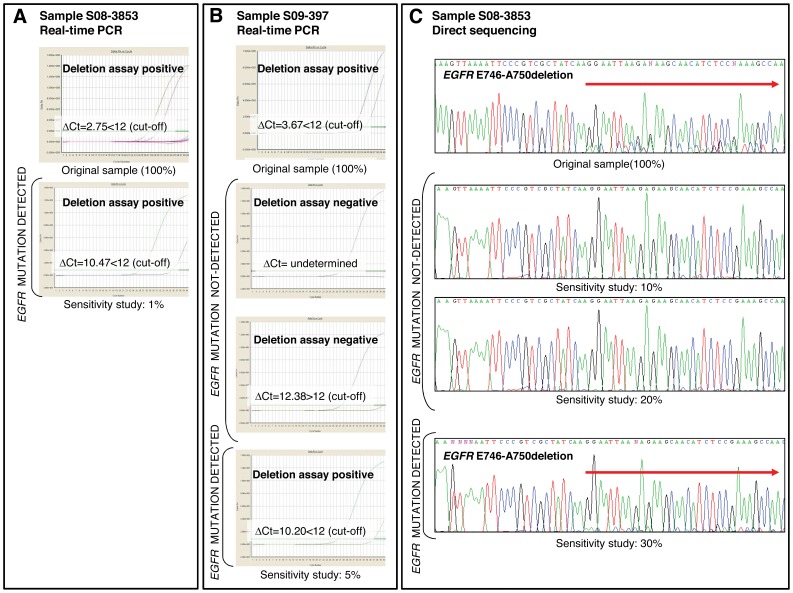
Limit of detection of the Therascreen EGFR Mutation Test Kit in comparison with direct sequencing. Serial dilutions of DNA from *EGFR* mutant and wild-type FFPE tumours were used to compare the relative sensitivities of both methods. (*A*), the Therascreen EGFR Mutation Test Kit was able to detect an *EGFR* mutation when the DNA from the mutant tumour represented 1% of the total DNA in half of the analyzed tumours. Sample S08-3853, which harbours an exon 19 deletion, is shown as an example. (*B*), the Therascreen EGFR Mutation Test Kit was able to identify *EGFR* mutation in a 5% dilution of the total DNA in all the tumours analyzed. Sample S09-397, which harbours an exon 19 deletion, is shown as an example. (*C*), at least 30% mutant DNA was necessary in a background of wild-type DNA to detect *EGFR* mutations by direct sequencing in most of the analyzed tumours. Sample S08-3853 is shown as an example. Percentages indicate the proportion of DNA from a mutant tumour relative to DNA from a wild-type tumour. The ΔCt cut-off value to detect the presence of an *EGFR* exon 19 deletion is provided by the manufacturer. It is derived from cell lines and synthetic constructs.

The results of the LOD study with tumours are summarized in Tables 4, 5 and 6 and an example is depicted in Figure 2. The Therascreen EGFR Mutation Test Kit was able to detect the presence of *EGFR* mutations when mutant DNA, relative to wild-type DNA, represented 1% of the total DNA in half of the tumours tested positive by the kit. None of these tumours was characterized by a relevant lymphocyte inflammation. Interestingly, all of these tumours had a percentage of tumour cells ≥40% (median = 59%, mean = 60%, range, 40 to 85%). Using the Therascreen EGFR Mutation Test Kit, *EGFR* mutations were identified in a 5% dilution of the total DNA in all of the tumours. One of the tumours identified as mutant at this dilution had a relatively low percentage of tumour cells (∼30%) and macrodissection could not be performed as it was a bronchoscopic biopsy. Another one was characterized by a relevant desmoplastic stroma.

**Table 4:** Study of the LOD of *EGFR* Mutation Testing Using Tumours: Summary.

% mutant DNA relative towild-type DNA	Therascreen EGFR MutationTest kit (n = 18)§, *	% mutant DNA relative towild-type DNA	Direct sequencing (n = 19)§, *	
1%	9/18 (50%)	10%	1/19 (5%)	
		20%	6/19 (31%)	
3%	15/18 (83%)	25%	6/19 (31%)	
		30%	12/19 (63%)	
5%	18/18 (100%)	N.D.	7/19 (37%)	

N.D., not determined.

§ No adequate DNA quantity was available for sensitivity study for two of the *EGFR* mutant tumours included in the whole serie (exon 19 deletion).

* The Therascreen EGFR Mutation Test kit characterized one *EGFR* mutant tumour as wild-type (an insertion in exon 20 was identified by direct sequencing) because it is not designed to detect this type of insertion.

**Table 5:** Study of the LOD of *EGFR* Mutation Testing Using Tumours: Real Time PCR.

	Therascreen EGFR Mutation Test kit (n = 18)§, *		
% mutant DNA relative to wild-type DNA	Exon 19 deletion (n = 11)	L858R (n = 5)	Exon 20 insertion (n = 2)
1%	8/11 (73%)	0/5	1/2 (50%)
3%	10/11 (91%)	4/5 (80%)	1/2 (50%)
5%	11/11 (100%)	5/5 (100%)	2/2 (100%)

N.D., not determined.

§ No adequate DNA quantity was available for sensitivity study for two of the *EGFR* mutant tumours included in the whole series (exon 19 deletion).

* The Therascreen EGFR Mutation Test kit characterized one *EGFR* mutant tumour as wild-type (an insertion in exon 20 was identified by direct sequencing) because it is not designed to detect this type of insertion.

**Table 6:** Study of the LOD of *EGFR* Mutation Testing Using Tumours: Direct Sequencing.

	Direct sequencing (n = 19)§, *		
% mutant DNA relative to wild-type DNA	Exon 19 deletion (n = 11)	L858R (n = 5)	Exon 20 insertion (n = 3)
10%	0/11	1/5 (20%)	0/3
20%	3/11 (27%)	1/5 (20%)	2/3 (67%)
25%	3/11 (27%)	1/5 (20%)	2/3 (67%)
30%	7/11 (64%)	2/5 (40%)	3/3 (100%)
N.D.	4/11 (36%)	3/5 (60%)	0

N.D., not determined.

§ No adequate DNA quantity was available for sensitivity study for two of the *EGFR* mutant tumours included in the whole series (exon 19 deletion).

* The Therascreen EGFR Mutation Test kit characterized one *EGFR* mutant tumour as wild-type (an insertion in exon 20 was identified by direct sequencing) because it is not designed to detect this type of insertion.

Direct sequencing was only able to detect the presence of an *EGFR* mutation in one of the tumours analyzed when mutant DNA represented 10% of the total DNA. Interestingly, this sample was a surgical specimen and had the highest percentage of tumour cells of the whole series (∼95%), therefore macrodissection was not performed. In most cases (63%), it was necessary to have at least 30% mutant DNA in a background of wild-type DNA in order to detect *EGFR* mutations by direct sequencing. However, it is important to note that direct sequencing was unable to detect the presence of *EGFR* mutations in seven tumours when mutant DNA represented ≤30% of the total DNA. Significantly, one of the tumours for which it was not possible to establish the sensitivity limit by sequencing had the highest lymphocyte inflammation of the whole series (75–80%). Another such tumour was the sample that performed poorly in the LOD analysis with the kit (5%) due to the relatively low percentage of tumour cells (∼30%).

If we consider the results of the LOD study according to mutation type, it is important to note that, for detecting the L858R point mutation in exon 21, a greater proportion of mutant DNA relative to wild-type DNA was required for both direct sequencing and real-time quantitative PCR. With the latter method, none of the L858R mutations was detected at a 1% dilution of the total DNA, while >70% of the deletions in exon 19 were identified at this dilution. In the case of direct sequencing, we could not establish the limit of detection for 60% of the L858R point mutations when mutant DNA relative to wild-type DNA represented ≤30%, while the number of samples with deletions in exon 19 for which there was no conclusive sensitivity limit was only about half.

Immunohistochemical expression of *EGFR* mutant-specific antibodies (E746-A750del and L858R) was evaluated in whole sections of 89 tumours with available tissue after molecular analysis (89 out of 136, 65.4%). Accordingly to direct sequencing analysis, these tumours included 70 *EGFR* wild-type and 19 *EGFR* mutants: 11 with exon 19 deletions, of which nine with E746-A750del and two with complex deletions, L747-A750>P and L747-P753>S; five with L858R; and three with exon 20 insertions. In addition, there was the result of the analysis with the Therascreen EGFR Mutation Test kit for 84 of these tumours, including 16 of the 19 tumours with mutation in *EGFR* previously indicated. As described earlier, DNA was not available for additional studies of two tumours with deletion in exon 19. One of the tumours with insertion in exon 20, detected by direct sequencing, produced a negative result with the kit (Table 7).

**Table 7:** Immunoreactivity of E746-A750del- and L858R-Specific Antibodies.

	IHQ							
*EGFR*	E746-A750-specific antibody			L858R-specific antibody			Direct Sequencing	Therascreen EGFR Mutation Test
	Intensity	%	H Score	Intensity	%	H Score		
Exon 20 mutation	0	0	Negative	N.A.	N.A.	N.A.	V769_D770insertion	Insertion
	0	0	Negative	0	0	Negative	V769_D770insertion	Insertion
	0	0	Negative	0	0	Negative	D770_n771insSVD	WT
Exon 21 mutation	0	0	Negative	0	0	Negative	L858R, T790M	L858R, T790M
	0	0	Negative	3+	90	Positive	L858R	L858R
	0	0	Negative	0	0	Negative	L858R	L858R
	0	0	Negative	3+	80	Positive	L858R	L858R
	0	0	Negative	0	0	Negative	L858R	L858R
Exon 19 mutation	3+	100	Positive	N.A.	N.A.	N.A.	E746-A750deletion	N.D.
	3+	100	Positive	N.A.	N.A.	N.A.	E746-A750deletion	N.D.
	2+	100	Positive	N.A.	N.A.	N.A.	E746-A750deletion	Deletion
	2+	100	Positive	0	0	Negative	E746-A750deletion	Deletion
	2+	50	Positive	0	0	Negative	E746-A750deletion	Deletion
	2+	80	Positive	0	0	Negative	E746-A750deletion	Deletion
	2+	100	Positive	0	0	Negative	E746-A750deletion	Deletion
	2+	90	Positive	0	0	Negative	E746-A750deletion	Deletion
	0	0	Negative	0	0	Negative	E746-A750deletion	Deletion
	0	0	Negative	0	0	Negative	L747-A750>P deletion	Deletion
	0	0	Negative	0	0	Negative	L747-P753>S deletion	Deletion

WT, wild-type.

N.A., not available for immunostaining evaluation.

N.D., no adequate DNA quantity available for real-time quentitative PCR analysis.

Comparison of the results of the analysis of mutations in *EGFR* using IHC with the results obtained by direct sequencing, and by the Therascreen EGFR Mutation Test kit, appears in Tables 8 and 9. All of the tumours characterized as wild-type by direct sequencing or real-time quantitative PCR were negative for staining with both antibodies. The expression of the antibody directed against the E746-A750 deletion was positive in eight of the nine tumours in which the aforesaid alteration was identified by direct sequencing. None of the tumours characterized as presenting a complex deletion in exon 19, different from the usual 15 nucleotides in E746-A750, showed positivity for this antibody. The Therascreen EGFR Mutation Test kit does not allow distinction between the different types of deletions in exon 19. As such, on comparing the results of the immunohistochemical analysis of this antibody with the results of the method based on real-time quantitative PCR, it became clear that the antibody detected the presence of the mutation in six of the nine mutant tumours analyzed. With regard to the L858R point mutation, of the five mutated tumours identified using direct sequencing or quantitative PCR, only two demonstrated positivity for the specific antibody. None of the tumours with insertion in exon 20 was positive for either of the two antibodies, which confirms the specificity of this approach.

**Table 8:** Comparison of the Results of the *EGFR* Mutation Analysis Between Immunohistochemistry and Direct Sequencing.

		Direct sequencing						
		E746-A750 deletion	Complex exon 19 deletions	L858R	Exon 20 insertions	Wild-types	Total	
IHC E746-A750 deletion	Positive	8	0	0	0	0	8	
	Negative	1	2	5	3	70	81	
	Total	9	2	5	3	70	89	
IHC L858R	Positive	0	0	2	0	0	2	
	Negative	8*	0*	3	2*	68*	81*	
	Total	8*	0*	5	2*	68*	83*	

* Immunohistochemistry staining with L858R-specific antibody was not evaluable in total of the tumours included in the study of *EGFR* mutations using PCR based methods.

**Table 9:** Comparison of the Results of the *EGFR* Mutation Analysis Between Immunohistochemistry and Therascreen EGFR Mutation Test Kit.

		Therascreen EGFR Mutation Test				
		All exon 19 deletions	L858R	Exon 20 insertions	Wild-types	Total
IHC E746-A750 deletion	Positive	6	0	0	0	6
	Negative	3	5	2	68	78
	Total	9	5	2	68	84
IHC L858R	Positive	0	2	0	0	2
	Negative	8*	3	1*	68*	80*
	Total	8*	5	1*	68*	82*

IHC, immunohistochemistry.

* Immunohistochemistry staining with the L858R-specific antibody was not evaluable in the total of the tumours included in the study of *EGFR* mutations using PCR based methods.

**Figure 3:**
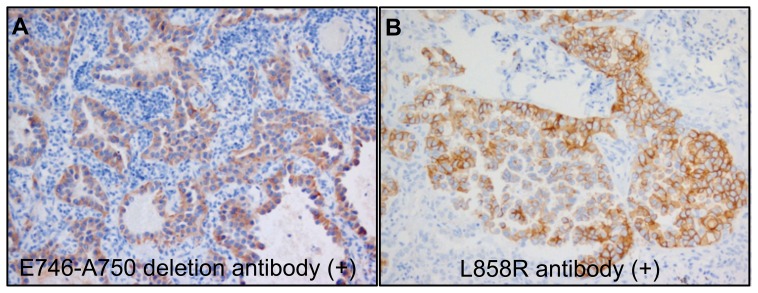
Immunohistochemical detection of (A) *EGFR* exon 19 E746-A750 deletion, and (B) exon 21 L858R point mutation. The images show a non-small cell lung carcinoma with an E746-A750 mutation stained with the specific anti- E746-A750del antibody (200x), and a non-small cell lung carcinoma carrying the L858R mutation stained with the specific anti-L858R antibody (200x).

The staining intensity was moderate to strong in all positive cases (Table 7 and Figure 3). Furthermore, all positive cases for IHC exon 19 had diffuse staining, while exon 21 positive tumours were always heterogeneous. Similarly, cross-reactivity of the antibodies was not observed and no tumour showed positivity for both.

In relation to the immunohistochemical study, the sensitivity and specificity of the two antibodies used, as well as their PPV, NPV and accuracy, are presented in Table 10. The specificity of the two antibodies was 100%, both when comparing the results of staining with the results obtained by direct sequencing and by real-time quantitative PCR. In terms of sensitivity, it is notable that the antibody directed against the E746-A750 deletion presented sensitivity close to 90% for detecting this deletion when compared with the results obtained by direct sequencing. Nevertheless, this sensitivity fell to approximately 73% on consideration of the total deletions in exon 19 detected by direct sequencing. Similarly, on comparing the results obtained with the kit, it is not possible to differentiate between the different types of deletions identified in *EGFR:* the sensitivity of the specific antibody in E746-A750 deletion was equally low. In addition, the sensitivity of the antibody directed against L858R point mutation reached only 40% when comparing the results of staining with those of sequencing or those obtained using the kit. On overall consideration of the two antibodies used in the study of mutations in *EGFR* by IHC and the results of sequencing, the sensitivity and specificity for the detection of mutations recognised by the antibodies were 71.4% and 100% respectively. Nevertheless, the sensitivity would decrease to almost 50% if the other mutations identified in *EGFR* were taken into account, including the complex deletions in exon 19 and the insertions in exon 20. This is the case if we compare the results of the immunohistochemical study with those of the Therascreen EGFR Mutation Test kit.

**Table 10:** Detection Capabilities for the *EGFR* Mutant Specific Immunohistochemistry *versus* Direct Sequencing and Therascreen EGFR Mutation Test kit.

		Direct sequencing							
IHC	*EGFR* mutations	Sensitivity (%)		95% CI	Specificity (%)	PPV (%)	NPV (%)	Accuracy (%)	
E746-A750	E746-A750 deletion	88.9		82.3–95.4	100	100	98.8	98.9	
E746-A750	All exon 19 deletion	72.7		63.5–82	100	100	96.3	96.6	
L858R	L858R	40		29.5–50.5	100	100	96.3	96.4	
E746-A750 + L858R	E746-A750 deletion + L858R	71.4		61.6–81.2	100	100	94.4	95.1	
E746-A750 + L858R	All mutations	46.7		35.9–57.5	100	100	89.3	90.2	
		**Threrascreen EGFR Mutation Test kit**							
**IHC**	***EGFR*** ** mutations**	**Sensitivity (%)**	**95% CI**		**Specificity (%)**	**PPV (%)**	**NPV (%)**	**Accuracy (%)**	
E746-A750	All exon 19 deletion	66.7	56.6–76.7		100	100	96.2	96.4	
L858R	L858R	40	29.3–50.7		100	100	96.2	96.2	
E746-A750 + L858R	All exon 19 deletion + L858R	53.8	42.9–64.8		100	100	91.8	92.5	
E746-A750 + L858R	All mutations	50	39–64.8		100	100	90.4	91.2	

IHC, immunohistochemistry.

CI, confindence interval.

PPV, positive predictive value.

NPV, negative predictive value.

## Discussion

The study of *EGFR* mutations in patients with lung carcinomas is complicated in the real clinical world by two interconnected factors. (a) Often we only have access to limited samples. *EGFR* mutation detection may be one test among several [27]–[30]. As such, there is no consensus on how to prioritize the different assays that are frequently performed in different laboratories: H&E and IHC for an accurate histological subtyping, FISH for *ALK* translocation, PCR for *EGFR* mutations, etc. This is a matter that needs to be carefully addressed as the number of lung targeted therapies increases (F. Lopez-Rios, unpublished data). (b) Seven years after the importance of determining the mutational status of *EGFR* in lung cancer was first demonstrated, there is still no standardized approach to performing this mutational analysis. A wide variety of methods, some of them laboratory developed tests, have been applied to *EGFR* mutation analysis including PCR-restriction fragment length polymorphism analysis, PNA-LNA PCR clamp, mutant-enriched PCR, dHPLC and COLD-PCR. Nevertheless, direct sequencing is still considered as the gold standard. Some of these assays are more sensitive than direct sequencing and could therefore overcome the limitations stated above. However, they are not suitable for routine clinical use for various reasons such as cost, complexity, long turnaround time, low specificity or unavailability [5], [31]–[34]. In summary, they would be more appropriate in very demanding scenarios, such as with a relatively young patient, an aggressive tumour or few treatment options, where we need to use a very fast and reliable assay.

The prevalence of mutations in *EGFR* identified (∼15%) was similar to that described in earlier studies conducted on advanced NSCLCs [34]–[36]. The deletions in exon 19, and the L858R point mutation in exon 21, are the most frequent. This is in line with what has been previously described both in Spain and elsewhere [23], [37].

With regard to the pre-analytical phase, in addition to the relative tumour content in the sample, the quality and quantity of the DNA extracted are also significant factors which can potentially affect the study of mutations. Our study demonstrates that it is possible to obtain DNA of sufficient quality to perform mutational studies of the *EGFR* gene, even when the parameters used to process the samples are unknown. However, it must be recognized that less DNA was recovered from small biopsies (bronchoscopic biopsies and CNBs). Nevertheless, there should be emphasis on the development of PCR approaches which use very little starting material, i.e. as few sections as possible, and which amplify smaller-sized fragments, thus guaranteeing that products are obtained even if the DNA is sparse or is particularly fragmented [33], [38], [39]. In addition, it is important to note that an insufficient yield in DNA extraction, which has been shown to be common in cases of small biopsies, can give rise to artefacts [39]–[41].

In assessing different analytical options, the LOD and the biological sensitivity of the methodology for studying *EGFR* mutations must be considered. Firstly, it is necessary to be aware of the LOD as this may relate to the minimum percentage of tumour cellularity necessary to detect mutation and may be considered a measure of the analytical sensitivity of the testing method. To the best of our knowledge, there are few studies that have compared different methods of studying the mutational status of *EGFR* [4], [5], [10]–[12], [42]. While the use of direct sequencing has begun to be questioned recently, it is worth noting that it remains the reference technique for the study of mutations in *EGFR* and in other genes despite its higher LOD, as confirmed by our analysis. However, the results obtained from sequencing are good: only ∼2% invalid test rate and 100% concordance with the results obtained by the Therascreen EGFR Mutation Test kit, taking into account the mutations covered by the kit. These results must be considered in the context of a laboratory with rigorous morphological control and which uses a highly prestigious core facility. Interestingly, our results show that if direct sequencing is performed with excellent quality, it is a reliable method for detecting *EGFR* mutations. Nevertheless, it is important to emphasise that the kit allowed characterization of one of the tumours which could not be assessed by direct sequencing. As such, it represents a good methodological approximation for the analysis of samples with poor quality of DNA.

The LOD of the Therascreen EGFR Mutation Test kit was 5% for all the tumour samples. As we have demonstrated, this LOD is much lower than that obtained by direct sequencing. Our experimental data agree with the clinical data of the BR.21 trial. A re-analysis with the Therascreen EGFR Mutation Kit found 7% more *EGFR* mutations in cases which were initially wild-type or could not be assessed with sequencing [44]. To the best of our knowledge, our study is the first experimental investigation of LOD of this kit that uses real clinical cases, contributing data which was lacking in the literature [45]. When compared with the majority of LOD studies of *EGFR* testing modalities, which have been conducted with cell lines or plasmid DNA [4], [6], [10], [46]–[48], the value of the approximation followed in this study is clear in reflecting the nature of the samples that are handled in a diagnostic laboratory. Moreover, the results obtained with commercially *EGFR* genetically defined standards were similar to those obtained by performing serial dilutions. This validates the latter approach to establishing the LOD.

The need for sensitive methods for the study of mutations in patients with lung carcinoma is especially relevant given that the percentage of cellularity of many of the samples presented for analysis, mainly small biopsies, can be a limiting factor after the use of classificatory IHC [49], [50]. The pathologist must evaluate the sample available, not only to determine the tumour percentage but also to assess the presence of fibrosis or lymphocytic infiltrate as these can also affect the sensitivity of the determination and the decision about which methodology to use [5], [22], [32]. In our series, several cases were paradigmatic in this regard although the analysis of the influence of the histological parameters in the detection of *EGFR* mutations was not statistically significant (data not shown). Compared with the PCR-based approaches, IHC represents an alternative for samples with a very low proportion of tumour [11]–[13], [15]. In the light of the above, it is evident that a collaborative effort between clinicians and pathologists is critical in ensuring the quality of *EGFR* testing.

The need for a highly sensitive method of analysing mutations also appears to be justified when we consider the question of the intra-tumour heterogeneity of the molecular alterations. If the quantity of cells with the genetic alteration were low, it is possible that the mutation would not be detected, even in the presence of a proportion of tumour cells appropriate to the LOD of the method used. As such, the staining observed for the antibody directed against the L858R mutation appears to confirm the heterogeneity of, at least, this *EGFR* alteration. This could explain the lower sensitivity of this antibody in comparison with that directed against the E746-A750 deletion. Equally, the results obtained in the LOD study would confirm these observations, as a greater relative proportion of mutated DNA was necessary for detecting the L858R mutations with the two PCR-based methods. The heterogeneity of the mutations in *EGFR* would be a factor to consider not only when selecting the method of characterization for the alteration, but also when assessing the different levels of response to ITKs and in determining whether the presence of a small number of mutated alleles could also be related to the response to the drug in question [5], [20], [21], [51], [52]. Interestingly, some of the studies cited were performed with the same methodologies as those presented here [20], [21], [52].

Secondly, as stated above, it would be necessary to consider biological sensitivity. Are the methods which detect the most common mutations sufficient or should we aspire to analyze all possible genetic alterations with a sequencing technique? The use of a commercial kit or IHC would be limited as these only detect the mutations for which they have been specifically designed: primers or antibodies, respectively. As such, they would give a false negative result for the other mutations, as happened in one of our cases. Furthermore, they do not permit differentiation between the different types of deletions and insertions characterized in *EGFR*. The clinical implications of many of the less frequent mutations remain to be determined [37], [53], [54].

Finally, the increasing demands in turnaround times give rise to the need for a method of analysis that offers an accurate result in as short a time as possible. Direct sequencing is an accessible technique but it requires experience to interpret the results and it involves multiple stages which slow the response time. Use of commercial kits gives greater speed and the possibility of automating the interpretation of results [33]. However, the technical requirements and cost make this an even less popular option [38], [39]. IHC is very accessible and can be automated, giving a rapid result, in as little as three hours using real-time IHC platforms such as the Benchmark XT (Ventana Medical Systems Inc.) (F. López-Ríos, unpublished data). Nevertheless, the potentially poor inter-observer and inter-laboratory agreement of IHC should always be taken into account [12].

While a literature review is difficult due to the different pre-analytical (tissue microarrays *versus* whole sections) and analytical (IHC devices) conditions, some comments are useful [11]–[21]. Not every author has found exclusive staining with the two antibodies [11], [13]. Cases of heterogeneity and false positives have been described with both [12], [13], [15], [18]. In this regard, the perfect specificity in our series strengthens the case for the algorithm proposed by Brevet et al [12].

In summary, we have presented a realistic comparison of different *EGFR* testing approaches. The LOD of the real-time PCR method was lower than that of direct sequencing. The mutation specific IHC produced excellent specificity. It is necessary to have methods available that give us access to results rapidly and accurately without depleting the sample and thus preventing accurate histological classification or the study of other biomarkers. We need to be aware of how the methods that we use perform in reality, above all when we have quantification of the *EGFR* mutations in mind. The biomarker which represented a shift in the world of lung targeted therapies must continue to lead the way.
